# Psychometric properties of the Greek Diabetes Treatment Satisfaction Questionnaire

**DOI:** 10.1186/1477-7525-10-17

**Published:** 2012-02-01

**Authors:** Nick Kontodimopoulos, Eleni Arvanitaki, Vassilis H Aletras, Dimitris Niakas

**Affiliations:** 1 Faculty of Social Sciences, Hellenic Open University, Patras, Greece; 2 "Ag. Varvara" Health Centre, Heraklion, Greece; 3 Department of Business Administration, University of Macedonia, Thessaloniki, Greece

**Keywords:** diabetes, DTSQ, treatment satisfaction, validity, reliability, Greece

## Abstract

**Objectives:**

Measurement of treatment satisfaction in diabetes is important as it has been shown to be associated with positive outcomes, reduced disease cost and better health. The aim of this study was to assess the construct validity and internal consistency reliability of the Greek version of the Diabetes Treatment Satisfaction Questionnaire (DTSQ).

**Methods:**

A sample of type II diabetes patients (N = 172) completed the DTSQ status version, the SF-36 health survey and also provided data regarding treatment method, clinical and socio-demographic status. Instrument structure, reliability (Cronbach's a) and construct validity (convergent, discriminative, concurrent and known-groups) were assessed.

**Results:**

The DTSQ measurement properties were confirmed in the Greek version with confirmatory factor analysis (CFA). Scale reliability was high (Cronbach's a = 0.92). Item-scale internal consistency and discriminant validity were also good, exceeding the designated success criteria. Significant correlations were observed between DTSQ items/overall score and SF-36 scales/component scores, which were hypothesized to measure similar dimensions. Known groups' comparisons yielded consistent support of the construct validity of the instrument.

**Conclusions:**

The instrument was well-accepted by the patients and its psychometric properties were similar to those reported in validation studies of other language versions. Further research, incorporating a longitudinal study design, is required for examining test-retest reliability and responsiveness of the instrument, which were not addressed in this study. Overall, the present results confirm that the DTSQ status version is a reasonable choice for measuring diabetes treatment satisfaction in Greece.

## Background

Diabetes is a major cause of morbidity and mortality and the prevalence of the disease has reached epidemic proportions, with the global number of people with diabetes projected to rise from approximately 170 million in 2000 to approximately 370 million in 2030 [1]. About 90-95% of all cases are type 2, also known as adult-onset diabetes [2]. Diabetes is further burdened with an increased risk of complications, which have important effects on patients' quality of life as well as socio-economic implications [3]. Overall, diabetes affects various domains of functioning and well-being and people with diabetes generally report worse health status and other outcomes than those without [4-6]. Severe dietary restriction, self administration of medications, routine blood glucose monitoring, and exercise demands are some of the ways in which diabetes can impact on quality of life.

Treatment satisfaction is defined by the individual's view of treatment and outcomes [7] and is particularly important for the acceptance of, and compliance with, treatment regimens [8]. Numerous health organizations have included measurement of patient satisfaction in projects designed to improve quality of care [9,10], which is evaluated by three equally important measures: structure, process and outcomes. Treatment satisfaction is included in the process component and is used as an important indicator of quality of care [10]. In diabetes, it has been shown that increased treatment satisfaction is associated with other positive outcomes [11], reduced disease cost [12-14], and improved health status [11,15,16].

The Diabetes Treatment Satisfaction Questionnaire (DTSQ) [17,18] is a globally accepted and used instrument to evaluate treatment satisfaction in diabetes mellitus. It has been recommended by the World Health Organisation and the International Diabetes Federation as useful in assessing outcomes of diabetes care [19] and has been used in many previous studies [11,15,20]. Given its widespread acceptance as a valid measure of treatment satisfaction in diabetes, the purpose of this study was to evaluate the psychometric properties of the original (status) version of the Greek DTSQ.

## Methods

### Instruments

The DTSQ was designed to make an assessment of total diabetes treatment satisfaction, treatment satisfaction in specific areas and perceived frequencies of hyperglycemia and hypoglycemia. It consists of eight items rated on a 0-6 scale. Six items specifically address: satisfaction with current treatment (item 1), treatment convenience (item 4), flexibility of treatment (item 5), understanding of diabetes (item 6), recommending treatment (item 7) and continuing treatment (item 8). The scores are summed into an overall satisfaction index ranging from 0 (very dissatisfied) to 36 (very satisfied). Items 2 and 3 evaluate the perceived frequency of hyper- and hypoglycemia respectively, with the scale reversed and lower scores indicating reduced hyper or hypoglycaemia. The DTSQ has been used extensively in clinical trials and in routine clinical monitoring and has now been linguistically validated in more than 100 languages. It has been used to measure patient satisfaction with treatment and has proved to be highly sensitive to changes in treatment, e.g. from rigid to flexible insulin treatment [21], from tablets to insulin injections [22] and from one insulin regimen to another [23,24]. The Greek version of the DTSQ has been linguistically validated using standard and well recognised methods [25], and the instrument developers were actively involved from the back translations onward, commenting and discussing at each stage.

The SF-36 Health Survey includes eight dimensions: physical functioning, "role physical", bodily pain, general health, vitality, social functioning, "role emotional" and mental health [26]. Each dimension is scored on a 0-100 scale with 0 and 100 corresponding to worst and best health status respectively [27], and the eight dimensions can also be summarized in two summary scores of physical and mental health [28]. The instrument has been translated into Greek and its reliability and validity were established in a representative sample of adults living in the greater Athens area. It was found to have high internal consistency reliability, convergent and discriminative validity and able to distinguish between groups of respondents in the expected manner (known-groups' validity) on the basis of gender, age and socio-economic status [29]. Using the same sample, the eight-scale structure of the Greek version of the instrument has been confirmed as well [30]. The SF-36 has been shown to discriminate well between perceptions of people with or without one or more chronic diseases [31], and has been previously used in Greek studies of people with diabetes [32,33].

### Sample and data collection

The data were collected between November 2008 and February 2009. The sample consisted of type 2 diabetes patients living in areas served by the "Agia Varvara" Health Centre, located close to Heraklion on the island of Crete. Consecutive patients were approached by the same social worker and asked to participate in the study. It should be noted that most of the residents in this Health Centre's catchment area are older people and of relatively low educational status. The presence of the social worker, with whom the patients were familiar with, aimed to assist them in understanding the questions and to resolve any misconceptions. The survey included the SF-36 and DTSQ as well as socio-demographic and other diabetes-specific questions. Completion time was approximately 25 minutes and 172 out of 191 patients visiting the facility during the study period agreed to participate (90.1% response rate). The Health Centre's Review Board granted ethical approval for this study and all participants provided informed consent.

### Analysis

Descriptive statistics and response frequencies for each item were calculated in order to examine central tendency, variability and symmetry. Scale reliability was calculated using Cronbach's alpha and the 0.70 standard for group-level comparisons was adopted [34]. Confirmatory factor analysis (CFA) was used to confirm that the measurement properties of the original version of the instrument apply to the Greek version as well. Goodness of fit was examined via: i) χ^2^/df (chi-square/degrees freedom) with values of less than 3 indicating a good fit, ii) root mean square error of approximation (RMSEA) with values < 0.05 indicating a close fit and iii) comparative fit index (CFI) for which values > 0.9 indicate a good fit [35].

Item internal consistency, which is substantial when correlation between an item and its hypothesised scale (corrected for overlap) is > 0.40, and item discriminant validity which is successful when correlation between an item and its own scale is significantly higher (> 2 standard errors) than with other scales [36], were used to examine the hypothesised structure of the instrument. Correlations between DTSQ items and SF-36 scales were examined to assess convergent construct validity, based on substantial correlations of related items/scales reported in the literature. Specifically, positive correlations were expected between treatment satisfaction and domains of physical and mental health status [37,38]. Known groups' validity was assessed by testing the DTSQ's ability to discriminate between groups of patients known to differ. Gender, age, years with diabetes, BMI, HbA1c level, treatment method, comorbidities, macro- and microvascular complications were used as testing criteria, and differences were examined with *t*-test and ANOVA. According to the literature, lower treatment satisfaction was expected in females [15,38,39], in younger diabetics [11], in insulin-treated patients [15,38,40], in the presence of diabetic complications [11,15,38,40], and in patients with higher BMI [41] and HbA1c [11,17] values. Multivariate OLS regression identified independent sociodemographic, clinical and treatment-related variables associated with treatment satisfaction and perceived hyper- and hypoglycaemia. Probabilities < 0.05 were regarded as statistically significant. All analyses were performed using SPSS software, version 17.0 (SPSS Inc., Chicago IL).

## Results

The demographic, treatment and clinical characteristics of the participants are presented in Table 1. The mean age of the entire sample was 65.2 years and the majority was women. The mean duration of diabetes was 5.2 years and the dominant treatment method was oral hypoglycaemic agents (OHA). The most common comorbid conditions in this sample were hyperlipidaemia and hypertension, whereas a high portion of respondents also had arthropathy. The most prevalent diabetic complication was macrovascular heart disease. At least one microvascular complication (retinopathy, neuropathy or nephropathy) was present in 7.6% of the sample. Diabetic foot, present in 6.4% of the patients, was examined separately as it could be argued that it has some elements of both micro- and macrovascular damage.

**Table 1:** Sample characteristics (N = 172)

	**(Mean ± SD)**
	
Age (mean ± SD)	65.2 ± 13.1
Diabetes duration (mean ± SD)	5.2 ± 4.9
BMI (Kg/m^2^) (mean ± SD)	31.2 ± 5.9
	
	N (%)
	
Gender (female)	107 (62.2)
Recent HbA1c level	
< 6.5%	49 (28.5)
6.5-7.5%	74 (43.0)
> 7.5%	49 (28.5)
Diabetes control method	
Oral agents	137 (79.7)
Insulin	35 (21.3)
Comorbid conditions	
Hyperlipidaemia	95 (55.2)
Hypertension	79 (45.2)
Arthropathy	31 (18.0)
COPD	15 (8.7)
Macrovascular heart disease	63 (36.6)
Microvascular disease	13 (7.6)
Diabetic foot	11 (6.4)

Descriptive statistics for the eight DTSQ items are presented in Table 2. Response rates were very high throughout, providing evidence that items and response choices were clear and unambiguous. Furthermore, most response choices were used in all of the items. The mode, i.e. the most frequently occurring response, was 3 for all but one of the six satisfaction items. Mean and median satisfaction values were in close proximity (17.5 and 18.0 respectively) implying a fairly symmetrical distribution of responses around the mean value. Item 1 regarding satisfaction with current treatment and item 7 regarding recommending the current form of treatment to someone with the same kind of diabetes had the highest and lowest mean scores respectively, which is surprising as these two concepts -yet distinct- are related to each other. Ceiling effects were not serious, suggesting that the instrument could record improvements at follow-up.

**Table 2:** DTSQ item descriptive statistics

Item	Description	Mean (SD)	Median	Item response distribution (%)						
				
				0	1	2	3	4	5	6
	*Treatment Satisfaction*									
										
1	Current treatment	3.17 (1.03)	3.00	1.2	4.1	18.0	39.0	29.1	8.7	-
4	Treatment convenience	3.00 (1.00)	3.00	0.6	5.2	22.7	43.6	21.5	5.8	0.6
5	Treatment flexibility	2.93 (1.09)	3.00	2.3	5.8	25.0	36.6	23.8	6.4	-
6	Treatment understanding	3.03 (1.07)	3.00	-	7.0	23.8	38.4	20.3	10.5	-
7	Treatment recommendation	2.23 (1.20)	2.00	7.6	19.8	31.4	29.1	8.1	4.1	-
8	Treatment continuation	3.00 (1.09)	3.00	1.2	3.5	26.2	37.8	26.2	5.2	-
	DTSQ score	17.48 (5.54)	18.00							
	*Diabetes Management*									
										
2	Perceived hyperglycaemia	2.58 (1.48)	2.00	6.4	20.3	25.0	18.6	16.3	13.4	-
3	Perceived hypoglycaemia	3.53 (1.37)	4.00	1.7	8.1	14.5	18.0	26.7	30.2	0.6

The CFA showed a covariance of 0.39 between the two factors implying good discriminative validity. Factor loadings ranged between 0.67-0.87 for treatment satisfaction and 0.48-1.22 for diabetes management (Table 3). Goodness of fit indices were: χ^2^/df = 2.01, RMSEA = 0.08 and CFI = 0.97, which imply satisfactory or better model fit [42]. Internal consistency of the 6-item scale (excluding items 2 and 3) was high (a = 0.92) and no item appeared problematic as overall internal consistency did not increase after deleting any of the items. Item internal consistency was substantial as the correlations between items and the overall DTSQ score was strong (with the respective item excluded each time) and ranged between 0.70 and 0.81, thus clearly exceeding the > 0.40 limit. Item discriminant validity was successful as the correlations between each item and overall DTSQ were significantly higher by two standard errors or more than with items 2 and 3, thus confirming the hypothesised DTSQ structure.

**Table 3:** Factor loadings, internal consistency and item-scale correlations (N = 172)

Item	Description	Factor loadings (CFA)	Internal consistency reliability^1^		Item-scale correlations^2^		
			
			Overall	Item deleted	DTSQ score	Item 2	Item 3
1	Current treatment	0.77	0.92	0.90	0.76	-0.17*	-0.26***
4	Treatment convenience	0.83		0.90	0.78	-0.18*	-0.35***
5	Treatment flexibility	0.74		0.90	0.73	-0.18*	-0.32***
6	Treatment understanding	0.87		0.89	0.80	-0.08	-0.25***
7	Treatment recommendation	0.67		0.91	0.70	-0.01	-0.31***
8	Treatment continuation	0.87		0.89	0.81	-0.24**	-0.38***
							
2	Perceived hyperglycaemia	0.48	0.78	NA	-0.20*	-	0.64***
3	Perceived hypoglycaemia	1.22		NA	-0.38*	0.64***	-

^1 ^Cronbach's alpha

^2 ^Corrected for overlap

*P < 0.05, **P < 0.01, ***P < 0.001 according to Pearson's correlation

Evidence of convergent construct validity (Table 4) was obtained by examining correlations between DTSQ items and SF-36 scales which were assumed to be conceptually related based on findings from the literature. Positive and statistically significant (*P *< 0.001) correlations were found between the DTSQ score and SF-36 physical functioning, general health, vitality, social functioning and mental health scales, as well as with the physical and mental health component scores. As for the specific DTSQ items, satisfaction with current treatment (item 1), treatment understanding (item 6), treatment recommendation (item 7) and continuing treatment (item 8), were the most strongly associated with the SF-36 scales. All the above-mentioned correlations were moderate or better and Pearson's r typically exceeded 0.3. On the other hand, perceived frequency of hyperglycaemic episodes was inversely associated with the general health, social functioning, role emotional and mental health dimensions, and with the mental health component. Perceived hypoglycaemia was negatively (and significantly) linked to general health and social functioning.

**Table 4:** Pearson's correlations between DTSQ and SF-36 scales and summary components (N = 172)

DTSQ items and scale^1^	SF-36 scales								SF-36 components	
	
	PF	RP	BP	GH	VT	SF	RE	MH	PCS	MCS
Current treatment (item 1)	0.31***	0.29***	0.24***	0.46***	0.30***	0.33***	2.83***	0.29***	0.36***	0.32***
Perceived hyperglycaemia (item 2)	-0.17*	-0.12	0.01	-0.20**	-0.08	-0.21**	-0.16*	-0.19*	-0.09	-0.21**
Perceived hypoglycaemia (item 3)	-0.12	-0.11	-0.02	-0.18*	-0.07	-0.18*	-0.08	-0.07	-0.10	-0.10
Treatment convenience (item 4)	0.16*	0.12	0.11	0.36***	0.17*	0.19*	0.09	0.17*	0.20**	0.17*
Treatment flexibility (item 5)	0.14	0.09	0.06	0.34***	0.13	0.17*	0.15*	0.19*	0.14	0.21**
Treatment understanding (item 6)	0.30***	0.22**	0.21**	0.47***	0.32***	0.32***	0.18*	0.29***	0.33***	0.30***
Treatment recommendation (item 7)	0.27***	0.16*	0.17*	0.35***	0.26***	0.29***	0.12	0.24**	0.27***	0.24**
Treatment continuation (item 8)	0.28***	0.19*	0.18*	0.45***	0.31***	0.30***	0.14	0.29***	0.30***	0.29***
Overall score (treatment satisfaction)	0.28***	0.19*	0.20**	0.47***	0.27***	0.32***	0.17*	0.28***	0.31***	0.28***

^1 ^DTSQ overall satisfaction score is the sum of responses to items 1, 4, 5, 6, 7 and 8

*P < 0.05, **P < 0.01, *** *P *< 0.001

Abbreviations: PF - Physical Function, RP - Role Physical, BP - Bodily Pain, GH - General Health, VT - Vitality, SF - Social Functioning, RE - Role Emotional, MH - Mental Health, PCS - Physical Component Score, MCS - Mental Component Score

Univariate analyses revealed no correlation between gender and treatment satisfaction (Table 5). As for age, lower mean DTSQ scores were observed with increasing age although the differences showed only a trend to significance (*P *= 0.067). On the other hand, the association between treatment/clinical parameters and satisfaction was stronger as expected. Specifically, DTSQ scores were significantly lower (*P *< 0.001) in groups with higher HbA1c values (> 7.5%) and in patients taking insulin. Presence of macrovascular coronary disease was significantly related with lower treatment satisfaction (*P *< 0.05) and patients with foot ulcers were also less satisfied. Patients with at least one microvascular complication such as retinopathy, neuropathy or nephropathy or one comorbid condition such as hyperlipidaemia, hypertension, arthropathy or COPD had slightly lower DTSQ scores, however differences were not significant. Perceived frequencies of hyper- and hypoglycaemia showed similar relationships with HbA1c and treatment method as the overall satisfaction score. The same applied for macrovascular coronary disease. The most noteworthy difference was the pronounced gender effect as women reported less hyper- and hypoglycaemia than men (*P *< 0.05 and *P *< 0.01 respectively).

**Table 5:** Perceived treatment satisfaction, hyper- and hypoglycaemia by demographic and diabetes-related variables

Variable	N (%)	Overall Treatment Satisfaction		Perceived Hyperglycemia		Perceived Hypoglycemia	
		
		Mean	P-value	Mean	P-value	Mean	P-value
Overall	172 (100)	17.48	-	2.58	-	3.53	-
Gender							
Male	65 (37.8)	16.86	*0.258*	2.94	***0.013***	3.91	***0.005***
Female	107 (62.2)	17.85		2.36		3.30	
Age group							
< 55	33 (19.2)	19.42	*0.067*	2.67	*0.985*	3.64	*0.908*
55-64	38 (22.1)	17.95		2.58		3.47	
65-74	59 (34.3)	17.02		2.54		3.58	
≥ 75	42 (24.4)	16.17		2.57		3.43	
Years with diabetes							
1-3	86 (50.0)	18.00	*0.273*	2.38	*0.214*	3.42	*0.504*
4-9	55 (32.0)	17.42		2.78		3.58	
≥ 10	31 (18.0)	16.13		2.77		3.74	
BMI							
18.5-24.9	16 (9.3)	17.69	*0.918*	2.25	*0.232*	3.44	*0.578*
25.0-29.9	70 (40.7)	17.47		2.84		3.66	
30.0-34.9	47 (27.3)	17.06		2.34		3.57	
≥ 35.0	39 (22.7)	17.48		2.54		3.28	
HbA1c							
< 6.5% (good)	49 (28.5)	20.43	***< 0.001***	1.53	***< 0.001***	2.41	***< 0.001***
6.5%-7.5% (fair)	74 (43.0)	17.31		2.82		3.86	
> 7.5% (poor)	49 (28.5)	14.78		3.27		4.14	
Control method							
Oral agents	138 (80.2)	18.61	***< 0.001***	2.34	***< 0.001***	3.30	***< 0.001***
Insulin^1^	34 (19.8)	12.88		3.56		4.47	
≥ 1 comorbidity^2^							
Yes	135 (79.7)	17.16	*0.147*	2.58	*0.951*	3.54	*0.833*
No	37 (20.3)	18.65		2.59		3.49	
Coronary/vascular disease							
Yes	63 (36.6)	16.07	***0.012***	2.92	***0.021***	3.71	*0.180*
No	109 (63.4)	18.08		2.39		3.42	
≥ 1 microvascular complication^3^							
Yes	13 (7.6)	17.15	*0.828*	2.62	*0.931*	3.23	*0.417*
No	159 (92.4)	17.50		2.58		3.55	
Diabetic foot							
Yes	11 (6.4)	12.55	***0.002***	3.27	*0.108*	3.73	*0.622*
No	161 (93.6)	17.81		2.53		3.52	

^1 ^Approximately half the diabetics in this group take oral agents alongside insulin

^2 ^Including hyperlipidaemia, hypertension, arthropathy or COPD

^3 ^Including retinopathy, neuropathy or nephropathy

*P*-values derived from independent samples *t*-test or ANOVA

The significance of the above-mentioned relationships between treatment satisfaction and disease management was confirmed with multivariate OLS regressions (Table 6). In the case of overall DTSQ scores, age, higher HbA1c values, treatment with insulin and diabetic foot were identified as significant predictors of reduced satisfaction, with the model explaining an overall 28.5% of the variance. The two clinical variables, i.e. HbA1c values and insulin treatment were the only significant predictors of increased perceived hyper- and hypoglycaemia, with the regression models explaining 23.2% and 26.8% of the variance respectively.

**Table 6:** Multivariate OLS regression models

DTSQ	Predictors	Β	SE	t	P-sig	R^2^
Satisfaction	(Constant)	30.976	2.686	11.532	**< 0.001**	**0.285**
	Gender (Female)	0.011	0.765	0.015	0.988	
	Age^1^	-1.031	0.406	-2.540	**0.012**	
	Years therapy^1^	-0.399	0.514	-0.777	0.438	
	BMI class^1^	0.019	0.416	0.045	0.964	
	HbA1c^1^	-1.874	0.511	-3.668	**< 0.001**	
	Control method^1^	-4.586	0.997	-4.601	**< 0.001**	
	Comorbidity	-1.002	0.897	-1.117	0.266	
	Coronary/vascular disease	-0.340	0.840	-0.405	0.686	
	Microvascular disease	2.398	1.444	1.661	0.099	
	Diabetic foot	-3.190	1.558	-2.047	**0.042**	

Perceived Hyperglycaemia	(Constant)	0.944	0.740	1.275	0.204	**0.232**
	Gender (Female)	-0.254	0.211	-1.205	0.230	
	Age^1^	-0.121	0.112	-1.082	0.281	
	Years therapy^1^	0.198	0.142	1.397	0.164	
	BMI class^1^	-0.099	0.115	-0.864	0.389	
	HbA1c^1^	0.676	0.141	4.800	**< 0.001**	
	Control method^1^	0.709	0.275	2.583	**0.011**	
	Comorbidity	-0.047	0.247	-0.189	0.851	
	Coronary/vascular disease	0.384	0.231	1.660	0.099	
	Microvascular disease	-0.303	0.398	-0.762	0.447	
	Diabetic foot	0.233	0.429	0.544	0.587	

Perceived Hypoglycaemia	(Constant)	1.815	0.674	2.694	**0.008**	**0.268**
	Gender (Female)	-0.281	0.192	-1.463	0.145	
	Age^1^	-0.077	0.102	-0.757	0.450	
	Years therapy^1^	0.202	0.129	1.569	0.119	
	BMI class^1^	-0.115	0.104	-1.105	0.271	
	HbA1c^1^	0.698	0.128	5.444	**< 0.001**	
	Control method^1^	0.768	0.250	3.073	**0.002**	
	Comorbidity	0.043	0.225	0.190	0.850	
	Coronary/vascular disease	0.148	0.211	0.704	0.482	
	Microvascular disease	-0.646	0.362	-1.784	0.076	
	Diabetic foot	-0.265	0.391	-0.677	0.499	

^1 ^According to the groups designated in Table 5

## Discussion

The DTSQ status version has been linguistically validated into Greek according to procedures documented elsewhere [25]. The aim of this study was to collect evidence of the validity and reliability of this Greek version for use in studies with Greek samples. The expected added value to the existing international body of knowledge on the subject is the increased confidence in results from national studies employing the instrument and the potential for international comparisons. Although generic health status instruments (e.g. SF-36) have shown their validity in Greek samples of people with diabetes [32,33], and the DTSQ has been used in one previous Greek study [43], we are unaware of previous studies measuring the psychometric properties of any diabetes treatment satisfaction instrument in Greece, implying that this research can fill an existing void in this area.

The overall results for the psychometric properties of the instrument were good. The absence of ceiling effects, although a sign of good discriminative ability, is fairly surprising in the context of measurement of satisfaction, which is known to often lead to skewed distributions of responses. In this respect, our results are inconsistent with previous studies having suggested maximum or close-to-maximum DTSQ scores at baseline. One explanation for the relatively low satisfaction reported here (17.5 mean DTSQ score) may come from the poor health status of the sample, although it is impossible to support any casual relationships with the data. The SF-36 scale scores (not shown) were lower for physical functioning, general health, vitality (*P *< 0.001), mental health (*P *< 0.01), social functioning and pain (*P *< 0.05) than the respective general population scores reported in the instrument validation study [29]. There is strong evidence in the literature supporting the positive association between health status and diabetes treatment satisfaction [11,15].

A cultural effect, in this particular sample, might also exist and explain to some extent the absence of ceiling effects. The health care facility in this study is located in a semi-urban/rural area where patients are attended by a generalist and not a diabetic specialist. Although the literature shows similar satisfaction between diabetes patients of family practitioners and endocrinologists/internists [44], studies in Greece have shown higher satisfaction with health services in urban compared to rural health facilities [45]. Furthermore, our low satisfaction scores are probably not linked to the presence of the social worker during completion of the survey by the patients, as this would have been expected to generate higher scores due to the possibility of social desirability bias. In any case, ceiling effects provide little opportunity for registering improvement in satisfaction with treatment or strategy being assessed [46,47], and it was for this reason that the DTSQ change version was designed and developed, and proposed for use in intervention studies in addition to the DTSQ (status) [48].

The measurement properties of the instrument were confirmed with CFA and goodness of fit was demonstrated via three different indices for which values were within, or close to, the suggested limits. Scale internal consistency reliability was very high and exceeded the corresponding reliability coefficients reported in similar studies [41,49-51]. Item internal consistency and discriminant validity were good as well, providing evidence to confirm the hypothesised structure of the DTSQ and to suggest that the translation of the items and the response choices are appropriate. It should be noted that the cross-sectional design employed in the present study precluded the assessment of responsiveness of the instrument.

In diabetes, the measurement of health status is concomitant with assessments of satisfaction with the quality of care [37]. In the present study, significant correlations were observed between the DTSQ items and overall satisfaction and the SF-36 scales and component scores. This was expected since diabetes is a chronic disease with a wide range of associated disability and discomfort which is typically captured by health status instruments. Our results conform to the literature which shows that poorer treatment satisfaction is generally associated with worse perception of physical and mental well-being [15,41,49]. In one particular study employing the DTSQ and the SF-12 (a shorter version of the SF-36 for measuring health status), lower treatment satisfaction was correlated with worse self-rated mental and physical health status scores [38]. However, in another DTSQ validation study employing the SF-36, strong correlations were found between WHO-WBQ scales and the mental dimensions of the SF-36 questionnaire, but not between DTSQ and SF-36 scores, suggesting that additional data are required to determine the most appropriate health status instrument to be administered alongside the DTSQ [51]. Regarding the two perceived blood glucose control items, the correlations between perceived hyper- and hypoglycaemia and SF-36 were in accordance with results from a similar DTSQ validation study [41].

Known-groups' validity was supported by the presence of macrovascular complications (coronary and/or vascular disease) negatively affecting treatment satisfaction. The same applied for diabetic foot, and although the subsample with foot ulcers was quite small (N = 11), the DTSQ decrements were sufficiently large to provide statistical power for comparisons. These findings are consistent with the results of numerous previous studies having reported a negative effect of the presence of any diabetic complications [11,15,38,41,52]. Higher satisfaction was recorded from patients on oral agents, as opposed to insulin therapy. Apart from the obvious fact that injecting insulin is less comfortable than taking a pill, this particular result might be indicating that patients perceive insulin treatment as deterioration in their health status. It might also be that diabetic patients who need insulin have longer disease duration with more complications. Higher HbA1c levels were also linked to lower treatment satisfaction in the univariate analyses and were also significant predictors of satisfaction, hyper- and hypoglycaemia in the respective regression models. In any case, there is disagreement in the literature on the association of these parameters and satisfaction as some studies have demonstrated the existence of such a relationship [11,15,37], while others have not [49,50].

Gender, disease duration and obesity were apparently not related to treatment satisfaction, however the former was associated with perceived hyper- and hypoglycaemia, as men experienced episodes more frequently than women. Most studies have indicated lower satisfaction among women [15,49,51], although others could not find any gender effects [41,53]. Age was a borderline significant (*P *< 0.067) factor with a negative relationship with satisfaction, however it was a significant predictor in the regression model, in accordance with results reported elsewhere [41]. The role of obesity in treatment satisfaction is not clear and some studies have confirmed it as a disadvantage [11,51], whereas other have not [49,53]. In our study, obesity was a non-relevant factor for treatment satisfaction. It is worth mentioning that the mean BMI in the sample was 31.2 Kgr/m^2^, which is apparently high but not unusual in this type of population, as shown in a relevant Greek study [32]. Moreover, the prevalence of obesity in Greece is the highest among European countries, reaching 26.0% and 18.2% in men and women respectively [54]. Finally, no association between comorbidities and treatment satisfaction was found, despite the high prevalence of comorbidities in this sample. A possible explanation is that the most common comorbid conditions were hyperlipidaemia and hypertension, which are "silent" diseases.

A potential limitation of this study might arise from the DTSQ being completed in the presence of a social worker who the patients knew and felt comfortable with. This approach, as opposed to self-administration for which the instrument was designed, was adopted as it would otherwise be difficult for this aged and low educational status rural sample to successfully complete the survey. One might claim that this may have weakened the results on quality of completion, since the social worker could actively ask for responses, or even have led patients to be more sincere, as patients tend to give socially desirable responses so as not to criticize their treatment (or their physician) [55,56]. As previously mentioned, the absence of a ceiling effect and the low satisfaction scores recorded in this study imply that social desirability bias was not a problem.

## Conclusions

Internal consistency reliability and cross-sectional construct validity of the Greek DTSQ were satisfactorily demonstrated; however the cross-sectional design of the present study precludes examining test-retest reliability and responsiveness. Thus, a longitudinal study design could be considered to overcome these limitations, as well as to validate the present results. The instrument was well-accepted by the patients and its psychometric properties were similar to those reported in validation studies of other language versions. Overall, the results confirm that the DTSQ status version is a reasonable choice for measuring diabetes treatment satisfaction in Greece. Providing that future research addresses the aforementioned issues, as well as testing the DTSQ change version, results from Greek studies can be expected to add to the international body of knowledge on treatment satisfaction.

## Competing interests

The authors declare that they have no competing interests.

## Authors' contributions

NK was responsible for designing the study, analyzing and interpreting the data, and drafting the manuscript. EΑ was responsible for data collection and assisted the statistical analysis. VA revised the manuscript for intellectual content. DN was responsible for conception of the study. All authors have read and approved the final manuscript.
